# The Suspension of Treatments in ADAPT: Concerns beyond the Cardiovascular Safety of Celecoxib or Naproxen

**DOI:** 10.1371/journal.pctr.0010041

**Published:** 2006-12-22

**Authors:** John C. S Breitner, Barbara K Martin, Curtis L Meinert

Dr. Nissen's comments [1] on our paper [2] reveal a fundamental difference in his and our perspectives on the ethical conduct of clinical trials. While we agree with his central point that the ADAPT data do not permit definitive conclusions regarding the safety of naproxen or celecoxib, we doubt Dr. Nissen's conjecture that the ADAPT safety data could—or should—have been more interpretable had a different model of data review been applied to adverse events.

Safety data in ADAPT, as in most trials, were not collected to test hypotheses. Instead, they were collected to protect trial subjects from harm to the extent possible, and to ensure that the investigators and the funding agency fulfilled their important obligations on this point. Stopping a trial for benefit and stopping to avoid harm are, fortunately, not symmetrical occurrences: typically one stops to avoid harm when inference is far less certain than would be required to affirm benefit. This principle—a direct reflection of the trial investigator's ethical obligations to study participants—was especially important for ADAPT, a primary prevention trial with 7–10 years of planned interventions and no anticipated short-term benefits. For the same reason, the stopping rules mentioned by Dr. Nissen are typically applied to prevent a premature conclusion of benefit, not of harm.

To clarify, it was the ADAPT Steering Committee, and not the National Institutes of Health (NIH), that bore ultimate responsibility for the decision to suspend the ADAPT treatments. The suspension of celecoxib was a direct consequence of Pfizer's announcement on December 17, 2004, of termination of the APC and PreSAP trials, citing cardiovascular risks with celecoxib [3] even though, as our paper shows, the ADAPT data suggested little cardiovascular risk with celecoxib [2].

The decision to suspend treatment with naproxen was more difficult. As we told the Food and Drug Administration (FDA) Advisory Committee [4], the safety data “…. would not in themselves have led to a decision to suspend either treatment…[because of any] conclusion that this signal [with naproxen] was…sufficiently compelling or definitive to warrant a recommendation to suspend the treatment.” Yet, several operational issues would have made continuation of the naproxen arm difficult. These included a necessity to obtain revised consent from participants—hence, review of new consent forms by seven Institutional Review Boards—as well as a reluctance to imply, by continuing naproxen, that it was “safe” while celecoxib was not.

Both decisions were taken in consultation with the Chair of the trial's data monitoring committee and with program staff of the National Institute on Aging. But in any event, on December 23, six days later, the FDA put a Full Clinical Hold on ADAPT and other primary prevention trials that administered celecoxib. The procedures required for reinstatement of the ADAPT investigational new drug (IND) application would have taken months, and in the interim all treatments would have been suspended regardless of our earlier decision.

We concur with Dr. Nissen, nonetheless, about the lamentable and somewhat misleading publicity that surrounded our decision. On December 20, 2004, the NIH Director's office reviewed the preliminary ADAPT cardiovascular safety results and forwarded them to the leadership of the FDA. The NIH Deputy Director then convened a conference call with the ADAPT Study Chair (JCSB) and Director of the Coordinating Center (CLM) to announce that a press teleconference would be held in cooperation with the FDA later that day. While that press conference was in process, the Director's office issued the press release referenced by Dr. Nissen, after which the ADAPT Study Chair was asked to answer several reporters' questions. The ensuing press coverage is well summarized by Dr. Nissen.

We also lament the long delay in publication of the ADAPT cardiovascular safety results. In fact, with full acknowledgement of their limitations, we had submitted these results to no fewer than five other journals before they were accepted by *PLoS Clinical Trials.* Consistently, the reason for rejection was that the data were “not definitive” (as we had acknowledged) or that results ran counter to expectation. Notwithstanding the logistical difficulties cited, we were surprised to learn of the other journals' evident view that publication of the ADAPT safety results would have been justified only if the treatments had been continued until there was a clear demonstration of harm with naproxen.

We remain resolute in our convictions on the following principles:

1. Ultimately, it is the investigators who are responsible for the well-being of those studied in trials. They report to Institutional Review Boards; data monitoring committees do not.

2. One does not do trials to find “significant” or “definitive” evidence of harm, especially in long-term prevention trials with healthy people. The risk–benefit balance in such trials is especially vulnerable to unforeseen situations such as arose for ADAPT on December 17, 2004.

3. Before blaming anyone for the decision to suspend treatments in ADAPT, one should consider the “mother test.” Given what is now known, would you have advised your aging mother to remain a participant receiving treatments in ADAPT? No member of the ADAPT leadership could answer this question “yes.”

4. While almost all will agree that some data—especially those from a randomized experiment unlikely to be repeated—are better than none, most journal editors and reviewers seem paradoxically to prefer “significant” or “definitive” untoward results. We think this is unfortunate. We agree that these data should have been published long ago. We are grateful to the editors of *PLoS Clinical Trials* for taking us at our word and publishing them. 
